# Der lange Schatten der Hyperinflation: Oder: Die Sicherheitspräferenz der Deutschen und deren Folgen für Staatsfinanzen, Kapitalanlage und Unternehmensfinanzierung

**DOI:** 10.1007/s41025-020-00203-2

**Published:** 2020-11-10

**Authors:** Michael Hüther

**Affiliations:** Institut der deutschen Wirtschaft, Konrad-Adenauer-Ufer 21, Köln, 50668 Deutschland

**Keywords:** Sicherheitspräferenz, Schuldenbremse, Venture Capital, Innovationen, Unternehmerbild

## Abstract

Deutschland weist im Umgang mit finanziellen Fragen einige Besonderheiten auf. Dazu gehören die geringen Erträge der umfangreichen internationalen Anlagen dieser exportorientierten Volkswirtschaft, die auch Jahrzehnte nach dem ersten Befund unzureichende Bereitstellung von Risikokapital und die im Vergleich mit vielen vergleichbar entwickelten Volkswirtschaften sehr restriktive Regulierung öffentlicher Kreditaufnahme. Die drei Aspekte haben insofern ein gemeinsames Muster, als sie auf eine mangelnde Bereitschaft deuten, auf künftiges Wachstum zu setzen und damit auch eine Risikoakzeptanz deutlich zu machen. Wie kann diese hohe Präferenz für Sicherheit erklärt werden? Dafür wird in der Tradition von Geert Hofstede nach den prägenden kulturellen Haltungen gefragt und als historischer Hintergrund für Deutschland die zweifache Hyperinflationserfahrungen im 20. Jahrhundert identifiziert. Welche wirtschaftspolitischen Handlungsmöglichkeiten es trotz habitueller Fixierungen gibt, wird abschließend erörtert. Die Antwort liegt in einer Mobilisierung staatlicher Investitionen, einer Innovationsinfrastruktur und einem positiven Unternehmerbild.

## Abstract (en)

Germany has some peculiarities when it comes to dealing with financial issues. These include the low return from the extensive international investments out of this export-oriented economy, the inadequate provision of venture capital even decades after the first finding, and the very restrictive regulation of public debt compared to many similarly developed economies. The three aspects have a common pattern insofar as they indicate a lack of willingness to rely on future growth and to accept risks. How can this high preference for stability and security be explained? For this, in the tradition of Geert Hofstede, questions are asked about the formative cultural attitudes and the twofold experience of hyperinflation in the 20th century are identified as the historical background for Germany. What economic policy options there are despite habitual fixations will be discussed in conclusion. The answer lies in the mobilization of state investments, an infrastructure for innovation and a positive image of entrepreneurial spirit.

Eine Inflation ist ein Massen-Vorgang im eigentlichsten und engsten Sinne des Wortes. Die verwirrende Wirkung, die sie auf die Bevölkerung ganzer Länder ausübt, ist keineswegs auf den Moment der Inflation selbst beschränkt. Man kann sagen, dass es in unseren modernen Zivilisationen außer Kriegen und Revolutionen nichts gibt, das sich in seiner Tragweite mit Inflationen vergleichen lässt. (Elias Canetti 1983, S. 202)

## Drei Beobachtungen und eine Erklärung

(1) Deutschland ist bei allem Erfolg seiner Wirtschaftsordnung und seines Geschäftsmodells im internationalen Vergleich auffällig, wenn es um Wachstumszuversicht und darauf gerichtete Handlungsstrategien geht. Drei Beobachtungen in diesem Kontext legen die Frage nach ihrem inneren Zusammenhang nahe. Da ist erstens die Feststellung, dass die Deutschen ihr Vermögen sowohl im Inland wie im Ausland weniger ertragreich anlegen als andere (Hünnekes et al. 2019a; 2019b). Dazu fügt sich zweitens der seit langem für Deutschland evidente Befund, dass die Bereitstellung von Risikokapital insbesondere für die Wachstumsphase eines Start-up-Unternehmens immer noch ein beachtliches Problem darstellt (Albach 1983; Albach und Köster 1997). Drittens kommt die Besonderheit hinzu, dass die Deutschen eine im internationalen Vergleich recht strenge Kreditregulierung für die öffentlichen Haushalte präferieren, die mit der Schuldenbremse im Grundgesetz im Jahr 2009 verankert wurde (Haffert 2016; Hüther 2019a). Die große Finanz- und Wirtschaftskrise hat diese Entwicklung durch den sprunghaften Anstieg der Schuldenstandquote von 60 auf 80 % befördert, der exogene Schock der Covid-19-Pandemie hat daran trotz erneuter sprunghafter und massiver Neuverschuldung im Grundsatz nichts geändert, wie die Tilgungspläne ab dem Jahr 2023 signalisieren.

**Figure 1:**
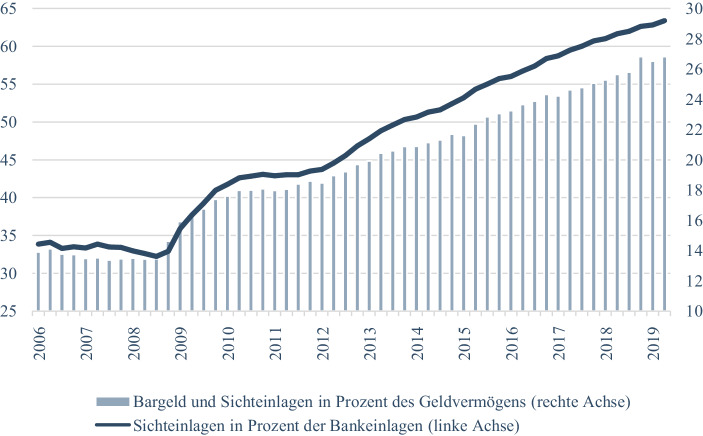

Alle drei Befunde haben insofern ein gemeinsames Muster und damit eine identische Ursache, als sie auf eine bestimmte Haltung und Risikobereitschaft in der deutschen Gesellschaft zurückzuführen sind, die im Vergleich zu anderen Ländern, beispielsweise in der Gruppe der G7, bemerkenswert ist. Die mangelnde Bereitschaft, auf künftiges Wachstum zu setzen und damit auch eine Risikoakzeptanz deutlich zu machen, geht mit einer hohen Präferenz für Sicherheit einher. In Deutschland stehen diese Werte ganz oben auf der Skala gesellschaftlich relevanter Positionen und Normen. Dies zeigt sich am Anlageverhalten in Zeiten persistent niedriger Zinsen, das sich durch robuste Anteile risiko- und renditearmer Vermögensarten auszeichnet^1^ (Abb. 1), und zwar auch auf Seiten institutioneller Investoren: hier ist im europäischen Vergleich die Verlustaversion und die Sicherheitspräferenz am größten (Union Investment 2016). Dazu passt, dass trotz vergleichsweiser geringer Rendite die deutschen Anleger besonders zufrieden sind, also nicht damit hadern, dass ihre ausgeprägte Risikoaversion einen hohen Preis in Form entgangener Erträge hat (Das Investment 2017).

(2) Diese Befunde sind grundsätzlich nicht neu. Bereits vor der globalen Finanz- und Wirtschaftskrise des Jahres 2009 wurde die Risikoaversion der Deutschen als „pathologisch“ bezeichnet (Frankfurter Allgemeine Zeitung 2005). Das spricht dafür, dass die Wurzeln weit zurückreichen.

- Ein Argument ist die bei aller berechtigten Kritik *verlässlich ausgebaute Altersvorsorge*, die in Deutschland gerade im Vergleich mit angelsächsischen Ländern die Anreize für private Vorsorge lange Zeit gemindert und die Illusion risikofreier Anlage genährt hat (Fohlin 2016, S. 17 f.). Das führt dazu, dass bei Analysen zur Vermögensverteilung Deutschland wie andere Staaten mit gut ausgebauter Alterssicherung (wie z. B. die skandinavischen Länder) eine relativ hohe Konzentration aufweist (Niehues 2018). Hinzu kommt, dass die in Deutschland besonders beliebte Lebensversicherung über Jahrzehnte steuerlich präferiert worden ist, was die Option risikoarmer Anlage für das Alter in den Augen der Bürger weiter gestärkt hat.
- Traditionell schwach ist in Deutschland die *Aktienanlage* ausgeprägt, weil von der Finanzierungsseite her seit langem andere Wege dominierten. Eine substanzielle und nachhaltige Schwächung der Aktienmärkte verband sich mit der Änderung der Eisenbahnfinanzierung am Ende des 19. Jahrhunderts. Zunächst hatte der Eisenbahnbau hierzulande wie weltweit die Aktienkultur belebt, da er von privaten Unternehmen getragen wurde und die Entstehung von Aktienbanken als Finanzierungsvehikel beförderte; Eisenbahnen sollten nicht aus dem Staatshaushalt finanziert werden. Als aber die Eisenbahnen von den Staaten im Deutschen Reich bis zum Jahr 1910 weitgehend verstaatlicht wurden, sind Aktien durch (Staats‑)Anleihen ersetzt worden. Damit wurde die öffentliche Wahrnehmung der Aktie hierzulande deutlich geschwächt (Breitfeld 1985). In den USA wurden die Bahnen von Anfang an in Form von Aktiengesellschaften geführt, auch wenn der Staat den Bau erheblich förderte (nach neueren Untersuchungen bis zu 40 % der Baukosten; Dobbin und Dowd 2000).
- Wie keine andere westliche Gesellschaft haben die Deutschen im 20. Jahrhundert zweimal eine galoppierende *Hyperinflation* erlebt, die das private Leben nahezu unbeherrschbar hat werden lassen und den öffentlichen Raum gemeinsamen Gestaltens der Lebensbedingungen in Frage stellte. Der fundamentale Mangel an Verlässlichkeit ist als Bedrohung im kollektiven Gedächtnis der Deutschen unverändert prägend; der Zerfall der Mittelschicht und die Hoffnung auf eine neue Mitte in der zweiten deutschen Demokratie stehen dafür ebenso wie die normative Positionierung deutscher Eliten wie deutscher Ökonomen in Bezug auf die makroökonomische Steuerung (Taylor 2013; Hayo und Neumeier 2016; Redeker et al. 2019).

(3) Es gibt offenkundig gesellschaftlich relevante Einstellungen und Verhaltensweisen, die kulturell codiert sind und bestimmte Traditionen reflektieren. Geert Hofstede hat dies in seinen umfangreichen empirischen Studien ermittelt und bebildert (Hofstede et al. 2002). Kultur ist für Hofstede „the collective programming of the mind distinguishing the members of one group or category of people from others“, und „national Culture cannot be changed, but you should understand and respect it“ (Hofstede Insights o.J.b). Die entsprechenden Studien haben seinerzeit viel Kritik hervorgerufen, doch in jedem Fall deutlich gemacht, dass jenseits der spezifischen Bestimmungsfaktoren einer Entscheidung (finanzielle Anreize, Rechtsstatus) die *kulturelle Codierung* dabei wirkt. Hofstede hat sechs Kulturdimension herausgearbeitet. Für den hier in Rede stehenden Kontext erweisen sich die folgenden Dimensionen als bedeutsam: (1) Unsicherheitsvermeidung (oder Risikoaversion als Abneigung gegen unvorhersehbare oder widersprüchliche und deshalb nur schwer auflösbare Entwicklungen) sowie (2) zeitlicher Planungshorizont der Gesellschaft.

- Die entsprechenden Ergebnisse belegen, dass die Vermeidung von Unsicherheit im Gesellschaftsvergleich für Deutschland einen relativ hohen Stellenwert hat. Hofstede verweist zur Erklärung darauf, dass fußend auf den Ideen der prägenden Philosophen Kant, Hegel und Fichte die deduktive Verständnissuche dominiert: „[…] be it in thinking, presenting or planning: the systematic overview has to be given in order to proceed. This is also reflected by the law system“. Und „Germans prefer to compensate for their higher uncertainty by strongly relying on expertise“ (Hofstede Insights o.J.a).
- Bezogen auf den zeitlichen Handlungshorizont indiziert der ermittelte Wert einen pragmatischen Ansatz. Damit verbinden sich eine hohe Neigung zu Sparsamkeit und zu Ausdauer in dem Erreichen von Zielen (Hofstede Insights o.J.a).

Diese Ergebnisse statten die anfängliche Vermutung, die Deutschen hätten eine hohe Präferenz für Sicherheit, Vorhersehbarkeit und Planbarkeit, mit empirischer Bestätigung aus – soweit dies für solche Fragen überhaupt möglich ist. Die These weiter zu verfolgen, in der kulturellen Kodierung liege auch der Grund für die identifizierten und auffälligen Befunde, erscheint jedenfalls danach mehr als sinnvoll. Zunächst wird ein vertiefter Blick auf die in Deutschland präferierte Finanzpolitik geworfen (Abschn. 2). Anschließend wird der Zusammenhang zwischen Hyperinflationserfahrung, Geldpolitik und Sparverhalten beleuchtet (Abschn. 3). Daran schließt sich eine Betrachtung der Besonderheiten in der Unternehmensfinanzierung hierzulande an (Abschn. 4). Es bleibt am Schluss die Frage, was zu tun ist (Abschn. 5).

## Die „schwäbische Hausfrau“ und die „schwarze Null“

(1) Emblematisch steht für die sicherheitsverpflichtete Haltung der deutschen Finanzpolitik die schwäbische Hausfrau, die durch Kanzlerin Merkel im Jahr 2008 sogar zum Idealtypus seriösen Wirtschaftens erkoren wurde (Merkel 2008): „Man hätte hier in Stuttgart, in Baden-Württemberg, einfach nur eine schwäbische Hausfrau fragen sollen. Sie hätte uns eine ebenso kurze wie richtige Lebensweisheit gesagt, die da lautet: ‚Man kann nicht auf Dauer über seine Verhältnisse leben.‘ Das ist der Kern der Krise.“ Was verbirgt sich hinter dieser Weisheit? Ist diese Weisheit für alle Wirtschaftsakteure – private Haushalte, Unternehmen und den Staat – gleichermaßen zu deuten und bedeutsam?

- Die *privaten Haushalte* erleben gewöhnlich im Lebenszyklus Phasen der Nettoverschuldung sowie Phasen des Nettovermögensaufbaus und müssen dies mit den unterschiedlichen Bedürfnissen während der Familiengründung, der Entwicklung von Familie sowie Haushalt in der Ausreifungsphase und im Rentenalter in Einklang bringen. Üblicherweise versuchen die Menschen, dem durch Tilgungspläne und Sparpläne Rechnung zu tragen, um im Alter ein auskömmliches Leben zu haben und den Kindern ein Erbe übereignen zu können. Hier ist die schwäbische Hausfrau ein passendes Vorbild, will man – anders gewendet – die Privatinsolvenz vermeiden und das Familienerbe verantwortlich weitergeben. Auf Dauer kann keiner über seine Leistungsmöglichkeiten und Vermögensverhältnisse leben.
- Bei *Unternehmen* sieht dies anders aus. Allein die Theorie des optimalen Verschuldungsgrads deutet darauf hin, dass hier eine andere Logik zum Tragen kommt. Unter den extremen Annahmen friktionsloser Kapitalmärkte, symmetrischer Informationsverteilung, fehlender steuerlicher Verzerrungen sowie Insolvenzkosten sind die Kapitalstruktur und damit der Verschuldungsgrad sogar neutral in Bezug auf den Unternehmenswert (Modigliani und Miller 1958), anders gewendet: Verschuldung beeinflusst den Wert einer Investition nicht. Die Kapitalstruktur gewinnt in dem Maße an Bedeutung, wie die genannten Annahmen nicht erfüllt sind. Aber auch dann ist die Fremdfinanzierung per se kein Ausdruck unsoliden Wirtschaftens. Es geht darum, den Schuldendienst aus dem Cashflow des Unternehmens zu leisten, was bei einer strategisch unterlegten Investitionsstrategie gut gelingen dürfte. Unternehmen sollten ausreichende Eigenmittel haben, um sich gegenüber Marktschwankungen absichern und langfristige Verpflichtungen (Betriebsrenten) bedienen zu können. Auf Dauer können Unternehmen sich demnach verschulden, wenn es zum Geschäftsmodell sowie den Ansprüchen der Anteilseigner passt und dadurch getragen wird.
- Der *Staat* wird anders als private Haushalte und Unternehmen als Institution mit Ewigkeitsgarantie betrachtet, in dem sich von Generation zu Generation der Übergang in der Verfügungsgewalt über den öffentlichen Kapitalstock vollzieht. Die extreme Langfristigkeit der Existenzunterstellung lässt sich am Kapitalmarkt an den Renditen für Staatsanleihen mit langen Laufzeiten erkennen. Wenn – wie erstmals Anfang August 2019 – dreißigjährige Bundesanleihen mit negativen Zinsen rentieren, dann bringt der Markt sogar ein besonders hohes Vertrauen in den Bestand des (deutschen) Staates zum Ausdruck. So geht es für Staaten nicht darum, Schulden definitiv zu tilgen, sondern um einen mit Blick auf die gesamtwirtschaftliche Leistungsfähigkeit, das Zinsniveau und die bestehende Schuldenquote, aber auch die politische Potenz auf Dauer tragfähigen Schuldenstand.

(2) Mit diesen Überlegungen zu den Sektoren Unternehmen und Staat kann die schwäbische Hausfrau, großgeworden im Lebenszyklus familiärer Hauswirtschaft, überhaupt nichts anfangen. Aus der Überschreitung der Perspektive einer Lebensbiografie ergibt sich aber überhaupt erst die Frage des fairen Ausgleichs zwischen verschiedenen Generationen. Das Gegenmodell zur schwäbischen Hausfrau, die zum Zeitpunkt ihres Todes alle Schulden getilgt hat und möglichst ein ansehnliches Nettovermögen hinterlässt, ist der Staat, der zu den Wirkungskontexten seiner Ausgaben eine adäquat fristenkongruente Finanzierung im Ausgleich zwischen den Generationen organisiert.

Doch geht es für den Staat nicht nur um wachstumstheoretische Einordnungen der Kreditaufnahmen, sondern ebenso um konjunkturelle Argumente. Die Frage, ob die Kreditfinanzierung staatlicher Ausgaben zugleich die private Nachfrage anregt und damit zur Glättung der Konjunktur beiträgt, hängt wesentlich davon ab, ob die privaten Haushalte davon ausgehen, dass die zusätzliche Verschuldung künftige Steuererhöhungen auslöst und deshalb die private Sparquote ansteigt (Barro-Ricardo-Äquivalenztheorem, Barro 1974). In diesem Fall wäre mit der Kreditfinanzierung wenig gewonnen. Allerdings wird dabei der angebotsseitige Effekt staatliche Kreditfinanzierung ausgeschlossen, und zwar sowohl in konjunktureller Hinsicht, was aber bei einer Verringerung der Unsicherheit über das Ausmaß rezessiver Entwicklung sowie über den mittelfristigen Potentialpfad erwartet werden kann, als auch mit Blick auf das Wachstum, was bei produktiven Ausgaben (Investitionen) positiv beeinflusst wird (zu den vorliegenden empirischen Studien vgl. Hüther 2019a). Die schwäbische Hausfrau muss mit Blick auf die staatliche Kreditaufnahme unter diesen kurzfristigen wie längerfristigen Bedingungen nicht sorgenvoll umhüllt sein.

(3) Es schließt sich die Frage an, wie im Handeln des Staates der faire Ausgleich zwischen den Generationen organisiert werden kann, genauer woran er sich orientieren sollte? Das ökonomische Argument des Pay-as-you-go impliziert eine faire Lastenverteilung dann, wenn jede Generation äquivalent zu ihrer Nutzenmehrung an der Finanzierung beteiligt wird. Die Kreditfinanzierung organisiert dies über die Zinszahlungen während der Laufzeit des dadurch ermöglichten Investitionsprojekts. Unterlassene substanzerhaltende Investitionen und eine nicht fristenkongruente Finanzierung, die Investitionen aus Steuermitteln oder nur mit kurzfristen Schuldtiteln ermöglicht, stellen gleichermaßen den fairen Ausgleich zwischen den zwei Generationen in Frage.

Aus Gerechtigkeitserwägungen lässt sich mit John Rawls argumentieren, dass eine faire Ordnung „die Freiheit und Unabhängigkeit der Bürger gewährleisten und fortwährend Tendenzen abschwächen sollte, die im Laufe der Zeit größere Ungleichheiten herbeiführen, welche sozialen Status und Vermögensverhältnisse ebenso betreffen können wie die Fähigkeit, politischen Einfluss geltend zu machen und verfügbare Chancen auszunutzen“ (Rawls 2006, S. 245). Rawls wirbt für ein angemessenes Sparprinzip, damit die Gesellschaft als „ein faires System der langfristigen Kooperation zwischen den Generationen“ funktioniert: „Es stützt berechtigte Beschwerden über unsere Vorgänger und berechtigte Erwartungen an unsere Nachfahren“ (Rawls 2006, S. 247).

Der zentrale Hinweis von Rawls liegt darin, im Blick auf die künftige Generation die der eigenen Generation vorangegangene nicht auszublenden. Wenn eine frühere Generation beispielsweise durch Krieg das Risiko eingegangen ist, den Kapitalstock zu zerstören, muss dies sich auch in den berechtigten Erwartungen der Nachfahren spiegeln, was beispielsweise die Hinnahme entsprechender öffentlicher Schulden für den Wiederaufbau des Kapitalstocks betrifft. Wenn klimapolitisch auch deshalb Handlungsbedarf besteht, weil frühere Generationen nicht oder nur unzureichend reagiert haben, dann sollte das heute insoweit über Steuern *und* Neukreditaufnahme finanziert werden, dass die heutige Generation nach Maßgabe ihrer Ressourcenübernutzung auf Konsum verzichtet und die künftigen Generationen für die Kompensation klimapolitischer Schäden mit bezahlen. Grundsätzlich entspricht diese Argumentation der „goldenen Regel der Finanzpolitik“.

(4) An diesem Sparprinzip sollten sich auch budgetpolitische Selbstbindungen messen lassen, indem sie Wirkungsasymmetrien vermeiden, weil Schulden, Steuerlasten, sozialstaatlicher Ausgleich und Investitionsmöglichkeiten nicht gesamthaft betrachtet werden. Die schwäbische Hausfrau jedenfalls bietet nicht die passende Orientierung für die Finanzierung der öffentlichen Haushalte. Allerdings hat sich diese Figur zur Ikone der deutschen Finanzpolitik entwickelt, deren Zuspitzung in der „schwarzen Null“ mündet und damit den jährlichen Haushaltsausgleich fordert („Die Null ist, ökonomisch gesehen, eine Zahl ohne besonderes Gewicht. Ihre eigentliche Bedeutung ist politischer Natur“‚ Haffert 2016). Zur Legitimation dieser kommunikativ gegenüber der verfassungsrechtlichen Lage verengten Zielsetzung der Finanzpolitik gehört auch die Erzählung, dass ohne Schuldenbremse alles aus dem Ruder zu laufen drohe wie zuvor; das freilich ist mehr Erzählung als Realität (Hüther 2019a).

An der dominanten polit-ökonomischen Erzählung der Begründung der Schuldenbremse hat sich auch nichts geändert als der Budgetsaldo des Staathaushalts im Jahr 2012 und der des Bundeshaushalts im Jahr 2014 ins Positive drehte. Die Überschüsse in den Budgets sind zur Selbstverständlichkeit geworden. Das ist für sich genommen nicht zu kritisieren, allerdings verstellt es den Blick auf eine nüchterne Einordnung des öffentlichen Kredits als Instrument der Finanzpolitik (Thöne 2005), vor allem der wachstumsorientierten Strategie. Es ignoriert die volkswirtschaftlichen Kosten, die damit verbunden sein können, wenn man diesen Finanzierungsweg sehr weitgehend tabuisiert. Und es verhindert die notwendige Analyse, was staatliche Kreditfinanzierung in Zeiten von Niedrigzinsen („low for longer“ oder „low for ever“ lautet mittlerweile die Frage zur künftigen Zinsentwicklung) und einem Zinssatz unterhalb der Zuwachsrate des BIP bedeutet, und zwar im politischen wie im öffentlichen Diskurs.

(5) Die Kunst guter Politik besteht darin, unter den jeweils obwaltenden gesamtwirtschaftlichen und budgetären Bedingungen instrumentell flexibel das langfristig fixierte Ziel zu verfolgen. Im Sinne des Rawlsschen Sparprinzips geht es um einen *fairen Ausgleich zwischen empfangenen Leistungen und zu tragenden Finanzierungslasten zwischen den Generationen*, darum in jeder Periode Freiheit und Unabhängigkeit der Bürger, eine hinnehmbare Verteilung sozialer Merkmale und Vermögen sowie einen wirksamen politischen Einfluss und die Wahrnehmung verfügbarer Chancen zu ermöglichen. Diesen Handlungszusammenhang durch eine Restriktion – wie die Schuldenbremse oder verengt die „schwarze Null“ – einzuschränken, kann rational nur mit der Erwartung begründet werden, dass dann negative Auswüchse (Gefährdung der Schuldentragfähigkeit) verhindert werden können und (neue) andere negative Effekte ausbleiben.

Das unterstellt, dass die Einschränkung eines prinzipiell unvollständigen Vertrages – als den man den Staatshaushalt (wegen Plan- und Genehmigungsvorbehalten sowie Umsetzungserfordernissen und Verpflichtungsermächtigungen, nur allgemeine Finanzierungsvorgaben (Non-Affektation)) verstehen kann – zu einem rational definierten vollständigen Vertrag führt, der eindeutig konsistente Ergebnisse zeitigt (Richter und Furubotn 2010). Doch dieser Vertrag bleibt unvollständig. Das äußert sich darin, dass zur Vermeidung negativer Kollateraleffekte nicht selten weitere Restriktionen vorgeschlagen werden, beispielsweise ein Deckel für die Sozialbeiträge oder die Unternehmenssteuerlast oder eine Investitionsquote. Darin drückt sich aus, dass eine Restriktion den unvollständigen Vertrag verändert und an anderer Stelle Strategieanfälligkeit begründet oder neue Restriktionen erfordert. Anders gewendet: Die Schuldenregel, die selbst der Sorge um den Parteienwettbewerb entspringt, verhindert diesen grundsätzlich nicht, indem sie den polit-ökonomischen Druck auf andere ausgabenseitige Budgetposten verschiebt oder Entlastungen bei Steuern und Abgaben verhindert. Diese Effekte, die sich in Veränderungen der Haushaltsstruktur manifestieren, werden in der Phase der Konsolidierung leicht übersehen. Grundsätzlich aber stellt sich die Frage, warum in der demokratischen Ordnung das Parlament sich freiwillig auf diese Weise selbst bindet.

- Rational kann dies als Absicherung der Abgeordneten gegenüber den Haushaltsspielraum strukturell, d. h. dauerhaft überfordernder Ansprüche der Wahlbürger eingeordnet werden (Buchanan 1984)
- Ebenso kann es darum gehen, im politischen Wettbewerb der Agenten (Parteien) um den Prinzipal (Wahlvolk) eine Bremse gegen einen überfordernden Ausgabenwettbewerb wirksam zu installieren (Nordhaus 1975).
- Schließlich kann die Überforderung des Staatshaushalts auf der Einnahmenseite ansetzen, wenn ein Wettbewerb um Steuersenkungen zu einer tendenziellen Verringerung der Staatstätigkeit (Staatsquote) mit dem Ziel des schlanken Staates führt (Persson und Svensson 1989; Haffert 2016).

Trotz der theoretischen Argumente für solche Regeln und der für diese Politik sowohl konservativ wie progressiv möglichen Rechtfertigung (Krebs 2019; Haffert 2016) bleibt die Frage, warum die Deutschen – ähnlich lediglich die Schweizer – eine besondere Präferenz für derartige restriktive Ausprägungen der Schuldenregulierung haben. Dies scheint in einem Mangel an Wachstumsfantasie und einer hohen Präferenz für Sicherheit begründet. Beides sind zwei Seiten einer Medaille. Die Sicherheitspräferenz führt zu einer Fokussierung des Erreichten, einer Stabilisierung der Gegenwart zur Verlängerung in die Zukunft. Eine selbstverständliche Erwartung auf Wachstum ist damit nicht so leicht kompatibel, denn Wachstum geht im Ökonomischen stets als Strukturwandel mit inkrementellen wie disruptiven Veränderungen einher und ist deshalb in seiner Qualität schwer prognostizierbar. Solche Haltung verweist auf historische Prägungen, für die „schwarze Null“ wird auf die Erfahrung der Staatspleiten nach den beiden Weltkriegen verwiesen (Haffert 2016). Und tatsächlich präferieren über zwei Drittel der Deutschen eine Verminderung der Staatsschuld – selbst in Krisenzeiten – und gut 60 % unterstützen die Schuldenbremse (Hayo und Neumeier 2016, S. 69 f.). Das gilt grundsätzlich auch in der Corona-Pandemie, die zu einer erheblichen Kreditaufnahme führte und die Schuldenstandquote allein im Jahr 2020 um 15 Prozentpunkte auf 75 % erhöhte. Mit der Schuldenbremse kann danach allenfalls der Bund zurechtkommen, keinesfalls die Länder sowie Kommunen, und erst recht ist die staatliche Investitionskrise darin nicht zu lösen (vbw-Studie 2020). Die robuste Akzeptanz der budgetpolitischen Beschränkung trifft sich mit der in Deutschland ebenfalls starken Präferenz für stabile Preise.

## Die Verantwortung der Geldpolitik und der Charme des Sparens

(1) In Deutschland lebt wie nur in wenigen Gesellschaften der Welt die Hyperinflation im kollektiven Gedächtnis fort, denn es hat als einziges industrialisiertes Land der Welt zwei große Inflationen und Währungsreformen im 20. Jahrhundert durchlebt (Feldman 1984). Die galoppierende Überraschungsinflation nach dem Ersten Weltkrieg (Papierwährung) und die Wertlosigkeit der Währung (Zigarettenwährung) nach dem Zweiten Weltkrieg haben sich normativ und habituell im Deutschsein niedergeschlagen.

Wenn die Geldentwertung täglich an Dynamik gewinnt, dann bedeutet dies, dass das Leben im hier und jetzt stattfindet, nur das tägliche Überleben zählt, dass jede Planung sinnlos und jedes Sparen irrational ist. Es kommt zu einer doppelten Entwertung: „Der einzelne fühlt sich entwertet … Die Masse fühlt sich entwertet. … Die Inflation hebt Unterschiede zwischen Menschen auf, die für die Ewigkeit geschaffen schienen, und wirft Leute, die einander sonst kaum gegrüßt hätten, … zusammen“ (Canetti 1983, S. 206). In einer solchen Lebenssituation dreht die Geschwindigkeit des Alltags sich in gleicher Tourenzahl wie die Inflation, Beständigkeit und Halt werden nicht gefunden, die Lebensverhältnisse vollends zerrüttet (Haffner 2000). Menschen und soziale Gruppen werden auf chaotische Weise neu- und angeordnet (Feldman 1984, S. 32). So war es während der Hyperinflation nach dem Ersten Weltkrieg, die aus Zwangswirtschaft und Reparationserfüllung mit Währungsverfall resultierte. Nicht anders war es grundsätzlich nach dem Zweiten Weltkrieg als zudem der gesamtwirtschaftliche Kapitalstock – öffentlich wie privat, in Form von Sachkapital wie Sozialkapital – weitgehend zerstört bzw. in seiner Funktionsfähigkeit eingeschränkt war. Zweimal im 20. Jahrhundert haben die Deutschen somit erlebt, wie erst mit einer Währungsreform die Stabilisierung der Volkswirtschaft und die Besänftigung der Gesellschaft gelang sowie die Handlungsfähigkeit der Politik verlässlich wiederkehrte.

(2) Die Währungsreform 1948 firmiert – in Ermangelung einer politischen überzeugenden Gründungsgeschichte – gar als wirtschaftlicher Gründungsmythos für das Provisorium Bundesrepublik, später verstärkt durch den „Ergänzungsmythos des ‚Wunders von Bern‘“ (Münkler 2009, S. 455 ff.): „Der gründungsmythische Kern der alten Bundesrepublik war nicht die politische Verfassung, sondern die wirtschaftliche Ordnung. Das ist bis heute der Fall, zumindest wenn man bedenkt, dass das Wirtschaftswunder mit der Entwicklung des Sozialstaats einherging“ (Münkler 2009, S. 458). Nach einem schwierigen Start schwenkte die bundesdeutsche Wirtschaft ab 1950 auf einen spektakulären Wachstumspfad ein, was dazu führte, dass die Währungsreform „im kollektiven Gedächtnis den Beginn eines wirtschaftlichen Wachstums markierte“ und damit zur gründungsmythischen Erzählung wurde, „die zu einer Identifikation mit dem neuen Staat beitrug“ und eine politische Selbstanerkennung verzichtbar erscheinen ließ; „aus der Opfergesellschaft des Krieges und der Entbehrungsgesellschaft der Nachkriegszeit wurde eine Konsumgesellschaft“ (Münkler 2009, S. 460 u. 464). Stabilisiert und gestärkt wurde dieser Mythos durch das allgegenwärtig persönliche Erleben und Erinnern; das Wirtschaftswunder war nichts Abstraktes, Theoretisches. All dies wurde weiterentwickelt im „Modell Deutschland“, mit dem Willy Brandt und Helmut Schmidt zu den Bundestagswahlen antraten und das Wohlstandsversprechen intensiver mit sozialem Ausgleich verbanden.

Das gesellschaftliche Korrelat zur bundesrepublikanischen Erfahrung anhaltender Wohlstandsmehrung war die Überwindung von bedrohlichen Randpositionen, die Zurückdrängung politischer Extreme im täglichen Leben und die Stärkung der Mitte (Münkler 2010, S. 205 ff.). Die von Helmut Schelsky so titulierte „nivellierte Mittelstandsgesellschaft“ stand für diesen Ausgleich und die Kraft der Mitte – politisch, ökonomisch und gesellschaftlich. Anders als in der ersten Hälfte des 20. Jahrhunderts erlebten die Menschen nun erstmals in der Breite, dass Industrie nicht zur Spaltung der Lebensbedingungen führte, sondern über die berufliche Bildung Aufstiegschancen glaubwürdig verhieß, die eine Verminderung der Einkommensspreizung beförderte. Der breite Aufstieg aus der Arbeiterklasse, die Verbürgerlichung der Lebensweisen in der Breite der Gesellschaft unterminierten die These des Klassenkonflikts; individuelle Leistung konnte nun auf verlässliche Weise für alle ertragreich sein, die es nur wollten (Münkler 2010, S. 217).

Es war der Ausdruck einer gemessen an den Erfahrungen der Weimarer Republik unglaublichen Stabilisierung und Stabilität aller Makrosysteme. Und so galt für Jahrzehnte nach dem Krieg: „Seit sich die Deutschen mit dem Mittelmaß abgefunden haben, sind sie selbst zur Ruhe gekommen und mit ihnen der ganze Kontinent“ (Münkler 2010, S. 219). Die Mitte als Maß der Orientierung – Maß halten und Mitte bewahren – bot die Möglichkeiten, sich den unvermeidbaren Veränderungen zu stellen. Denn „eine Gesellschaft könne sich umso mehr Modernisierung, lebenspraktische Differenzierung und Individualisierung leisten, je schwergewichtiger und selbstbewusster ihre Mitte ist“ (Münkler 2010, S. 223).

(3) Der Gewinn an Stabilität kann in seiner mentalitätsprägenden Kraft nur vor dem Hintergrund der – wie angedeutet – zersetzenden Kraft anhaltender, galoppierender Hyperinflation als gesellschaftliches Erleben verstanden und eingeordnet werden. In solchen Phasen werden die Möglichkeiten täglicher Routine und Verlässlichkeit zerrieben durch den Zwang, stündlicher Wertänderung durch neue Dispositionen Rechnung zu tragen. Versorgungsketten funktionieren nicht mehr, die Vorteil wirtschaftlicher Integration geht verloren und die Suche nach Sachwerten behindert das rationale Investieren. Besonders eindrücklich hat diese alltäglichen Folgen der Hyperinflation Sebastian Haffner (2000, S. 53, 57) geschildert: „Es kam das Jahr 1923. Dieses phantastische Jahr ist es wahrscheinlich, was in den heutigen Deutschen jene Züge hinterlassen hat …, jene hemmungslose zynische Phantastik, jene nihilistische Freude am ‚Unmöglichen‘ um seiner selbst willen, jene zum Selbstzweck gewordene ‚Dynamik‘. … Es war eine Lage, in der Geistesträgheit und Verlaß auf frühere Erfahrung mit Hunger und Tod bestraft, aber Impulshandeln und schnelles Erfassen einer neuen Lage mit plötzlichem ungeheurem Reichtum belohnt wurde. … Unter so viel Leid, Verzweiflung und Bettlerarmut, gedieh eine fieberhafte, heißblütige Jugendhaftigkeit, Lüsternheit und ein allgemeiner Karnevalsgeist“.

Diese Erfahrung war so überragend für die Zeitgenossen, dass sie auch in der Überlieferung die Bedeutung behalten hat und spätere Krisen in der Wahrnehmung dominierte oder gar überrollte. So zeigen aktuelle Befragungen, dass in Deutschland einerseits Hyperinflation und Weltwirtschaftskrise im Rückblick auf die Wirtschaftsgeschichte der Weimarer Republik verschmelzen und andererseits dieses Missverständnis „bei gut gebildeten und politisch interessierten Deutschen besonders ausgeprägt“ ist (Redeker et al. 2019, S. 3). Die Inflation ist – so zeigt der Befund – von überragender Bedeutung im kollektiven Gedächtnis und nicht die spätere Deflation mit Massenarbeitslosigkeit während der Weltwirtschaftskrise. Alles fügt sich im Rückblick zusammen, die „große Unordnung“ von 1919–1924 (Feldman 1997) ist der heuristische Anker für die Deutschen, wenn es um die Bewertung gesamtwirtschaftlicher Risiken und deren wirtschaftspolitischen Beantwortung geht. Selbst die Disinflation seit den frühen 1980er-Jahren und längere Zeiten sehr niedriger Inflationsraten haben nichts daran geändert, dass der Inflationsbekämpfung oberste Priorität eingeräumt wird; auch im Vergleich der europäischen Staaten zeigt sich diese extreme Fixierung in Deutschland auf die Preisniveaustabilität (Hayo und Neumeier 2016, S. 65 ff.).

(4) Der individuell basierte Befund – hier zitiert als Lebensbericht des jungen Sebastian Haffner über die wilden, zerrüttenden Jahre der Hyperinflation – hat in anderer Weise bei dem zeitgenössischen Soziologen Helmuth Plessner seinen Niederschlag gefunden. In der Analyse zu den „Grenzen der Gemeinschaft“ spiegelt sich die Inflation in der soziologischen Bestandsaufnahme. Denn nach der Überwindung der schlimmsten Verwerfungen im Jahr 1923 diagnostiziert Plessner als deutsches Problem die Vereinbarkeit von Idee und Wirklichkeit. Statt unbekümmert zur Tat zu schreiten und das Leben spielerisch zu nehmen, ist der Deutsche nun „schwer und über ihm wird alles schwer, heißt es bei Goethe (…) Der Deutsche ist stolz darauf, in seinen besten Männern das Gewissen der Welt zu sein, aber heißt das nicht auch für die anderen den Spielverderber zu spielen?“ (Plessner 1924, S. 20). Auf Prinzipien gegründete Stabilität sucht der Deutsche in der Gemeinschaft, eine scheinbar natürliche Ordnung der Lebensbezüge zwischen den Menschen, die auf Werten basiert, während hingegen die Gesellschaft als etwas Künstliches erscheint, in welcher der Umgang gewaltsam und anonym ist.

Das Erleben der Hyperinflation hat das Flüchtige, Unberechenbare der abstrakten Gesellschaft deutlich gemacht. Plessner argumentiert unabhängig von einer konkreten gesellschaftlichen und wirtschaftlichen Lebensordnung, er thematisiert grundsätzlich die Entwurzelung des Menschen unter den Bedingungen der anonymen Gesellschaft und der verzweifelten Suche nach Halt. Dabei erweise sich die Sachgemeinschaft als schwach, insofern sie auf abstrakte Ideen, Werten und Normen beruht, die leicht zu bezweifeln sind. Und so wird der Glaube aus Vernunft, dass es für alle Menschen eine gleich gute Lebensweise gäbe, schnell schwach, zumal nach dem Erleben einer Hyperinflation. Dadurch sind alle Gesetzmäßigkeit des normalen Lebens außer Kraft gesetzt worden, das Effizienzversprechen der modernen Wissenschaft wurde nachhaltig erschüttert. Dann kann es leicht passieren, dass bei der nächsten Verwerfung – wie der Weltwirtschaftskrise ab 1929 – der Glaube an die rationale Gestaltung der öffentlichen Aufgaben vollends schwindet. Anders gewendet: Die große Unordnung der Hyperinflation hat – so haben es angesichts der kurzen Phase der „goldenen Zwanziger“ die Zeitgenossen erlebt und so wirkt es bis heute fort – in Einheit mit der Weltwirtschaftskrise die gesellschaftliche Stabilität nachhaltig geschwächt.

(5) So wie nach dem Ersten Weltkrieg dessen finanziellen Verwerfungen und Folgen die Hyperinflation verursacht hatten, so war es nur zwanzig Jahre später der Zweite Weltkrieg zusammen mit der vorangegangenen Aufrüstung mit Lohn- und Preisstopp, die zu einer ähnlichen Zerrüttung der Lebensgrundlagen geführt hatten. Das Erlösende der Währungsreformen – Rentenmark zum 15. November 1923, Deutsche Mark zum 20. Juni 1948 – war offenkundig, doch erst mit dem Wirtschaftswunder nach 1950 entstand die Chance zur Mythenbildung. Entscheidend war eine Kombination ordnungspolitischer Entscheidungen, die dafür sorgten, dass Währungsreform und Leitsätze-Gesetz, Marshall-Plan und Londoner Schuldenabkommen ihre stabilisierenden Wirkungen entfalten konnten. So sehr hier Hoffnung als realistische gesellschaftliche Kategorie begründet wurde, so sehr stand dagegen als Menetekel die Periode von 1914–1924 („the great disorder“, Feldman 1997), die auch alle späteren Bemühungen wirtschaftlicher Stabilisierung in ein fades Licht rückte und als eine wesentliche Ursache der nachfolgenden Krisen des 20. Jahrhunderts erschien.

Daraus folgt, dass die Deutschen Stabilität mehr schätzen als unabsehbaren Wandel, vor allem die Stabilität des Geldwerts wird hoch bewertet, ohne sie ist alles nichts; die Vordenker der Sozialen Marktwirtschaft erhoben dies zum grundlegenden Prinzip. Die im internationalen Vergleich hohe Sparneigung verdichtet dies symbolhaft. Diese mikroökonomisch relevante Haltung reflektiert sich makroökonomisch in der Verfassung der Notenbank; die Erfahrungen mit der Hyperinflation führten nahezu selbstverständlich zu deren Unabhängigkeit, wie sie im Jahr 1957 mit dem Bundesbank-Gesetz verankert wurde und sich in eine stabile Wirtschaftsordnung mit hoher korporatistischer Prägung einfügte (Ritschl 2005). So wurde ein Bereich makroökonomischer Steuerung der parlamentarischen Verantwortung und Kontrolle entzogen; die Bundesbank ist nur ihrem gesetzlich definierten Mandat verpflichtet. Diese Delegation durch Schaffung eines Autonomiebereichs beruht auf dem historisch begründeten Misstrauen gegenüber Regierung und Parlament in Fragen des Geldes – finanzpolitisch wie geldpolitisch. Diese Einschätzung ist im Übrigen bis heute kein Gegenstand des Streits zwischen den Parteien im Deutschen Bundestag.

(6) Die spezielle Haltung der Deutschen ist nachhaltig wirksam, was sich insbesondere in der Kommentierung der europäischen Geldpolitik zeigt (Redeker et al. 2019). Dies sorgt zuweilen für Irritationen bei anderen europäischen Akteuren in der Geldpolitik. So klagte im Dezember 2013 Mario Draghi über Kritik aus Deutschland und sprach von „perverser Angst, dass sich die Dinge zum Schlechten entwickeln“; passiert sei aber das Gegenteil. „Die Inflation ist niedrig, und die Unsicherheit hat sich verringert.“ (Deutsche Welle 2013). Die Missverständnisse sind nur zu erklären aus der tiefen Verankerung unterschiedlicher Haltungen zum Geldwert und der dafür als passend erachteten Geldpolitik. Mit dem Euro machen die Deutschen die Erfahrung, dass eine bestimmte institutionelle Lösung noch keine Gewähr dafür bietet, dass die Geldpolitik unverdrossen der deutschen kulturellen Prägung Rechnung trägt. Die Sensibilität gegenüber der Konsumentenpreisinflation ist nicht überall gleich ausgeprägt, die Sorge vor dem Vermögensverzehr richtet sich dann auf andere Anlagekategorien. Wenn aber die Wohneigentumsquote in Deutschland aus diversen Gründen nur bei 43 % liegt, dann ist viel Raum für Sparen, der allerdings nicht sonderlich risikogeneigt ausgenutzt wird.

Vielmehr zeigt sich, dass die allgemeine Angst vor Unvorhersehbarkeit in Deutschland die Präferenz für „fixed income“ stärkt. So richtet sich die individuelle Vermögensposition der Deutschen ebenfalls mehr auf verlässliche Entwicklung und Stabilität als auf dynamische und volatile Wertentwicklung. Als typisch dafür kann die Regelung für die Riester-Rente gesehen werden, die einen jederzeitigen Kapitalerhalt vorsieht, was risikoreichere Anlageformen mit schwankenden Marktpreisen stark einschränkt. Die Bereitschaft, bei der Kapitalanlage Risiken einzugehen und auf einen Wachstumsgewinn als Return zu wetten, ist spiegelbildlich gering ausgeprägt. Diese Denkfigur ist nur in definierten Strukturen tragfähig, also in den Traditionen des Mittelstandes und vor allem der Familienunternehmen, wenn es darum geht, nachhaltig die Marktposition und die Vermögensentwicklung zu stabilisieren. Diese Denkfigur ist allerdings seit vier Jahrzehnten unter Druck geraten, ohne dass es zu Haltungsänderungen in der Breite der Bevölkerung gekommen wäre. Anders kann die besonders scharfe Kritik an der EZB, ihrer unkonventionellen Geldpolitik seit 2015 und den Niedrigzinsen nicht erklärt werden.

Dabei hatte bereits in den späten 1970er-Jahren mit dem Phänomen der Stagflation – als Folge des Zusammenbruchs von Bretton Woods und der ersten Ölverknappungen – der Mechanik der Wohlstandsmehrung über den Konjunkturzyklus hinweg ihre Verlässlichkeit verloren; erstmals war (begrenzte) Inflation nicht mehr als Preis höherer Wachstumsdynamik oder geringerer Arbeitslosigkeit vorübergehend als akzeptabel erschienen. Die stabilen 1980er-Jahre waren dann global durch Bemühungen der Disinflationierung geprägt und erweckten den Eindruck, dass trotz zunehmender Internationalisierung der Arbeitsteilung die alten Zusammenhänge wieder wirkten, der Beschäftigungsaufbau bei stabilem Preisniveau war beachtlich. In den 1990er-Jahren ging es um die Transformation der ostdeutschen Wirtschaft sowie deren Integration in den westdeutschen Ordnungsrahmen und die weltwirtschaftlichen Zusammenhänge. Doch selbst in dieser außergewöhnlichen Situation war es für die Deutschen völlig akzeptabel, dass die Bundesbank frühzeitig auf die damit verbundenen inflationären Schübe reagierte und den Diskontsatz im Jahr 1992 bis auf 8,75 % anhob.

## Die Finanzierung der Unternehmen: Was in Deutschland anders ist

(1) Die ausgeprägte deutsche Stabilitätskultur im Kontext von Staatsverschuldung und Inflation spiegelt sich, so die hier vertretene These, in der Finanzierungskultur für Unternehmen. Der Fixstern der deutschen Unternehmensgeschichte ist das Familienunternehmen in langer Tradition, das im Bereich der industriellen Fertigung eine besondere Weltmarktstellung erlangt hat und damit im Kern des deutschen volkswirtschaftlichen Geschäftsmodells – mittelständisch, industriebasiert, dienstleistungsergänzt, exportorientiert – steht (James 2006). In Deutschland sind 90 % der Unternehmen familienkontrolliert, und diese sind für 58 % der Beschäftigung verantwortlich (Stiftung Familienunternehmen). Familienunternehmen stehen dabei in besondere Weise für stabile Netzwerke, Traditionen und Langlebigkeit und offerieren somit Sicherheit. Diese besondere Struktur der deutschen Unternehmen ist damit verbunden, dass die Finanzierung über Aktien in viel geringerem Ausmaß der Fall ist als in den USA. Das hat historische Gründe.

In den letzten über zwei Dekaden haben die Unternehmen ihre Eigenkapitalausstattung spürbar erhöht, und zwar von gut 7 auf rund 25 %; die Finanzierung über Anleihen spielt keine große Rolle; bei langfristigen Finanzierungen sind die Banken unverändert der wichtigste Kreditgeber (Bendel et al. 2016). Insbesondere im Mittelstand zeigt sich eine hohe Robustheit der Finanzierungsstruktur: Die Eigenmittelquote lag im Durchschnitt der letzten Dekade bei 50 %, während auf Bankkredite 30 % entfielen und Fördermittel 13 % erreichten; 7 % blieb durchschnittlich für andere Finanzierungswege übrig^2^. Der Anstieg der Eigenmittelquote – aus einbehaltenden Gewinnen – setzt sich über alle (nichtfinanziellen) Unternehmen bis zum aktuellen Rand fort (Deutsche Bundesbank 2018): „Das Bild einer deutlichen Eigenkapitallücke zwischen kleinen und großen Unternehmen, auf das viele ökonomische Probleme des Mittelstands in der Vergangenheit regelmäßig zurückgeführt wurden, ist somit inzwischen weitgehend überholt. […] In der Gesamtschau bringen die Anpassungen in den Finanzierungsstrukturen wie auch der Liquiditätshaltung erhebliche Vorteile für die finanzielle Bestandsfähigkeit der nichtfinanziellen Unternehmen gegenüber exogenen Schocks mit sich und dürften zu einer höheren Krisenresistenz des deutschen Unternehmenssektors beitragen“ (Deutsche Bundesbank 2018, S. 72 f.).

**Figure 2:**
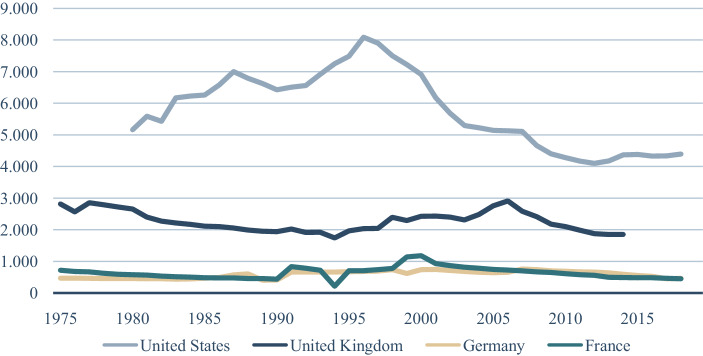

Die Aktienfinanzierung ist unverändert nachrangig, wenn man die Anzahl der gelisteten Unternehmen betrachtet (Abb. 2). Die Anzahl börsennotierter Unternehmen sinkt zwar auch in den angelsächsischen Volkswirtschaften, das dürfte dort aber mit einer höheren Private Equity Verfügbarkeit, einem durch die Digitalisierung getriebenen geringerem Kapitalbedarf mit einem geringeren Bedarf an Streuung zusammenhängen, während speziell in Deutschland eine geringere Gründungsaktivität als Ursache wirkt (Demary und Röhl 2017). Der Unterschied zwischen den angelsächsischen Volkswirtschaften und Deutschland ist markant; die Aktionärsquote liegt dort mindestens doppelt so hoch wie hierzulande. Die Anzahl der Besitzer von Aktien und Aktienfonds liegt hier bei gut 10 Mio., die anhaltenden Niedrigzinsen haben nur einen recht geringen Push-Effekt hin zur Aktienanlage (Deutsches Aktieninstitut 2019).

Die Präferenz der deutschen Privatanleger ist auf die Dividende gerichtet: Dabei gewichten „Privataktionäre positive Erfolgstrends stärker […] als negative. Insgesamt weisen Dividendenänderungen für die Anlageentscheidung – übereinstimmend mit der Untersuchung aus 2013 – die größte Relevanz auf, gefolgt von Gewinn- und Cashflowänderungen“ (Pellens et al. 2019). Und: „Die hohe Relevanz von Dividendenänderungen deckt sich auch mit der Entwicklung der Dividendenpräferenz von Privatanlegern. Auch wenn die Mehrheit der Befragten (45 %) nach wie vor ein tendenziell ausgewogenes Verhältnis von Dividenden und Kurssteigerungen bevorzugt, haben sich seit 2008 deutliche Verschiebungen in Richtung höherer Dividenden und geringerer Kurssteigerungen ergeben. Darüber hinaus zeigt das Antwortverhalten der Privataktionäre hinsichtlich der neu aufgenommenen Frage, ob sie Ausschüttungen in Form von Dividendenzahlungen oder Aktienrückkäufen präferieren, dass sie mehrheitlich (56 %) höhere Dividenden gegenüber Aktienrückkäufen bevorzugen“ (Pellens et al. 2019). Die Privatanleger hierzulande präferieren also vor allem die Dividendenzahlung, den sicheren Ertrag der Aktie im Hier und Jetzt, während Kurshoffnungen nur eine deutlich geringere Bedeutung haben^3^. Auch das spricht für Sicherheit als dominante Präferenz.

(2) Dabei ist es nicht so, dass deutschen Unternehmen kein Wachstum kennen – anders wäre der Erfolg der vergangenen Dekade nicht zu erklären, doch dies Wachstum ist vor allem eines, das über Differenzierung und Spezialisierung läuft, weniger über Skalierung; die Innovationen sind stärker inkrementell und weniger disruptiv. Im Strukturwandel hat sich dies bisher in einer beachtlichen Anpassungsflexibilität gezeigt, die das industriebasierte Modell – gerade auch im Ländervergleich – so profilieren konnte. Die gesamtwirtschaftliche Folge ist eine Erwerbsintegration, die mit 80 % der 15- bis 64-Jährigen einen historischen Höchststand und ein im Standortvergleich beachtliches Niveau erreicht hat.

„Among large, advanced economies, only Germany has managed to reverse the decline. German manufacturing value added has increased by 38 % since 1999, and it resumed strong growth after the Great Recession. Labor reforms in the early 2000s to freeze wages, promote job-sharing, and expand worker training helped restrain costs while preserving talent. High-quality products and a competitive currency helped German firms of all sizes gain global market share, creating a large and growing trade surplus“ (McKinsey Global Institute 2017, S. 29). Im Vergleich zu den Vereinigten Staaten zeigen sich ebenso kulturelle Prägungen, die auf beiden Seiten zu den strukturell und quantitativ unterschiedlichen Entwicklungen beitragen: „An ‚everyone for themselves‘ ethos can cause strains in a sector that combines inputs from multiple firms. In contrast to the institutional support enjoyed by Germany’s Mittelstand (medium-size firms), small and midsize US manufacturers typically lack financial, technical, and business development help. The German approach may not translate into the US context, but there are ideas to extract from it about the value of greater coordination. … The prime example of this model is the institutional support enjoyed by Germany’s Mittelstand (medium-size firms). While the German approach to cooperation and collaboration may not translate to the US context, it does offer some lessons about how coordination and scale can produce economic sustainability“ (McKinsey Global Institute 2017, S. 14, 58). Dazu gehören auch Unterschiede in den Bildungssystemen: „Apprenticeships that pay trainees while they learn on the job are widely available in countries such as Germany and Switzerland, and the model is finally gaining attraction in the United States“ (McKinsey Global Institute 2017, S. 16).

**Figure 3:**
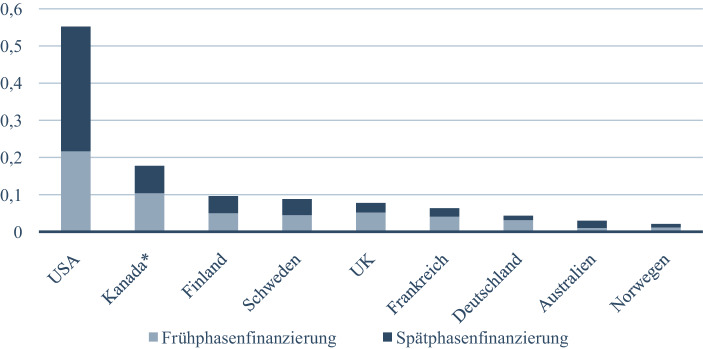

(3) Diese Hinweise sind deshalb bedeutsam, weil sie erkennen lassen, dass Volkswirtschaften sich in unterschiedlichen historischen Pfaden bewegen können (Hüther 2018), ohne damit insgesamt erfolgloser oder erfolgreicher zu sein als andere. Vielmehr erweisen sich die historisch definierten Strukturbedingungen als Differenzierungskraft. Freilich können solche Pfade in unterschiedlichen Perioden zu unterschiedlichen Ergebnissen führen. Derzeit wird vor allem darüber diskutiert, wie und mit welchen Chancen sowie Risiken der digitale Strukturwandel bewältigt und gestaltet werden kann. Dabei wird der deutschen Volkswirtschaft ein besonderer Nachteil attestiert: das Finanzierungssystem, genauer: die Bereitstellung von Venture Capital (Abb. 3). Hier wird schon traditionell zwischen den USA und Deutschland die „Venture Capital Divide“ identifiziert, wobei gilt „a complex of political, social, and economic factors—many dating back to institutions put into place in the 19th century—explains the evolution of venture capital over the post-war era“ (Fohlin 2016; so Albach 1983, S. 90 ff.).

Risikokapital (Venture Capital) steht dabei für eine Investition in besonders risikoreiche, weil innovative Firmen in ihrer Gründungsphase, bei der die Hoffnung auf „high growth and high returns“ zusammenkommt und eine enge Begleitung sowie Monitoring des Unternehmens durch den Investor üblich ist (Fohlin 2016, S. 3; Albach 1983, S. 70). Dahinter steht die Erkenntnis, dass „mit unterschiedlichen Risiken behaftete Investitionen […] nach unterschiedlichen Finanzierungstiteln“ verlangen. […] „Fehlende Finanzierungsmöglichkeiten für risikoreiche Investitionen bedeuten geringeres Wachstum, niedrigeren Beschäftigungsstand und Verminderung der internationalen Wettbewerbsfähigkeit“ (Albach 1983, S. 18 f.). Venture Capital ist gegenüber der Bankfinanzierung im Vorteil, weil es das Problem der asymmetrischen Informationsverteilung zwischen Unternehmen und Investor in der Frühphase einer Innovation respektive Unternehmensgründung besser bewältigen kann. Die enge Begleitung sichert nicht nur den notwendigen Informationsaustausch, sondern diszipliniert die Startup-Unternehmer im Umgang mit Risiken. Dabei wirkt sich auch aus, dass die VC-Firmen im Lebenszyklus der Startups unterschiedliche Finanzierungsinstrumente einsetzen und die Exit Option über Kapitalmärkte im Auge haben.

Genau entlang dieser Bedingungen zeigen sich die unterschiedlichen Bestimmungsfakten für die „Venture Capital Divide“ (Fohlin 2016, S. 11 ff.): Die Finanzintermediation in den USA ist geprägt durch die strenge Regulierung der Banken nach 1933 (Glass-Steagall-Act 1933, Banking Holding Company Law 1956), eine Deregulierung der Pensionsfonds für Risikokapitalanlage. Unterstützend wirkte u. a. seit 1953 die staatlicherseits eingerichtete „Small Business Administration“, aber ebenso Regierungsprogramme im Zusammenhang mit dem New Deal und der militärischen Forschung. Wichtig sind kulturelle Prägungen, vor allem eine aktive Unternehmenskultur, die das Scheitern nicht zum Desaster erklärt und immer offen ist für erneute Versuche. „Many of these factors worked in precisely opposite direction in Germany“ (ebd.).

(4) Es ist in Deutschland nicht gelungen, die große Dynamik unternehmerischer Gründung aus dem späten 19. Jahrhundert über die Weltkriege hinweg zu retten und zu transformieren; dabei beanspruchte Deutschland zurecht am Vorabend des Kriegsausbruchs 1914 „leadership in world innovation and production“ (Fohlin 2016, S. 24). Die Reichseinigung hatte die wirtschaftliche Entwicklung enorm begünstigt, dazu gehörte die Entwicklung der Universitäten, die Etablierung technischer Hochschulen, die Zusammenarbeit der großen Erfinder mit der akademischen Forschung und die Einführung der dualen Berufsausbildung (Hüther 2018); Deutschland war vor dem Ersten Weltkrieg führend in Bereich Unternehmensführung und der Rechnungslegung. Die Stärke der Unternehmenskooperation wurde durch Kartelle forciert, die in Deutschland besonders häufig, in den USA jedoch bereits seit Ende des 19. Jahrhunderts verboten waren. Gerade diese Konzentration verschärfte sich in Deutschland in der Periode der Weltkriege. Dies war vor allem der gezielten Kriegsvorbereitung im Dritten Reich (Etablierung des Vierjahresplans 1936) geschuldet, indem die Finanzierung über Aktien politisch grundsätzlich verpönt war.

Nach dem Zweiten Weltkrieg erwies sich dies als behindernd, wenn es darum ging neue Finanzierungswege zu eröffnen. Der besondere Schutz der Gläubiger und der relativ schwache Stand der Aktionäre wirkten sich stabilisierend auf die vorher etablierten Unternehmensnetzwerke der Industrie mit der Finanzwirtschaft aus. Das Informationsproblem zwischen Investor und Unternehmen wurde in diesen Netzwerken gelöst. Hinzu kam, dass nach dem Zweiten Weltkrieg der Wiederaufbau des zerstörten Kapitalstocks im Vordergrund stand und dafür Produktivitätsfortschritte zu organisieren waren, aber weniger in innovative Spitzentechnologie investiert wurde (Fohlin 2016, S. 19). „After 1913 Germany was thrown into a long period of chaos, beginning with World War I, continuing into the Weimar Republic, and culminating with the Nazi era and defeat in World War II. The cold war also played out most vividly on German soil“ (Fohlin 2016, S. 18).

(5) Die Venture Capital Divide ist in einem breiten thematischen Kontext verankert und steht damit nicht nur für die Entwicklung der spezifischen Risikokapitalfinanzierung in Deutschland, sondern sehr viel grundsätzlicher für die Finanzierungskultur. Die seit den 1980er-Jahren beklagte Risikokapitalschwäche (Albach 1983; Albach und Köster 1997) greift mit ihren Ursachen also ebenfalls weit zurück. Darin spiegeln sich nachfrageseitige und angebotsseitige Besonderheiten am deutschen Standort wider. Die Studienergebnisse von Hofstede (Hofstede et al. 2002) zur kulturell bedingten geringeren Risikoneigung der Deutschen lassen sich damit konsistent verknüpften, denn es passt zur Vermeidung von Unsicherheit, wenn die Finanzierung sich weniger auf grundsätzlich unvorhersehbares Marktgeschehen einlässt und sich stattdessen in etablierten Netzwerken bewegt. Diese später sogenannte „Deutschland-AG“ aus dem Netz von Industrieunternehmen, Banken und Versicherungen, die erst seit der durch die Unternehmenssteuerreform des Jahres 2000 ermöglichten steuerfreien Veräußerung von Kapitalanteilen aufgelöst wurde, bot genau den Rahmen, der mit Sicherheitspräferenz und Pragmatismus mentalitätsmäßig passend war.

Ebenso lässt sich das Anlageverhalten der Deutschen aus dieser Risikohaltung erklären. Denn der im internationalen Vergleich nicht nur marginal, sondern deutlich schlechtere Anlageerfolg – immerhin lag der durchschnittliche Ertrag der deutschen Auslandsanlagen 5 (3) Prozentpunkte unter dem vergleichbarer US- (europäischer)-Anlagen (Hünnekes et al. 2019a, S. 3) – findet seine Ursachen in einer entsprechenden Erklärung: „The overwhelming share of German foreign investments is located in other industrial countries with similar demographic profiles. […] the under-performance of German foreign investment is particularly pronounced for equity and foreign direct investments“ (Hünnekes et al. 2019a, S. 45). Risikoaversion und Sicherheitspräferenz drücken sich in der Wahl der Anlageländer aus, nicht aber in einem unterschiedlichen Risikoprofil bei der Auswahl der Einzelinvestments. Das entspricht der Systematik der Asset Allocation, nach der die Einzeltitelauswahl in einem Korb in ihrer Bedeutung für das Rendite-Risiko-Profil hinter der Makroauswahl zurückfällt.

(6) Während in angelsächsischen Ländern Stockoptions seit langem ein Element der Vergütung von Personen mit Führungs‑, Management- oder besonderer Ergebnisverantwortung sind, ist dies in Deutschland erst in den letzten zwei Jahrzehnten der Fall. Aktienoptionen sind Bezugsrechte auf Aktien, sie stellen eine besondere Form der Vergütung sowie auch eine attraktive Möglichkeit der Mitarbeiterbeteiligung dar. Der Schwerpunkt auf eine Vergütung durch Aktienoptionen und Bezugsrechten im angelsächsischen Raum kann als Reaktion auf das „principal-agent“ Problem verstanden werden. In den USA führte es zu einem Vergütungsmodell, welches die Interessen der Manager mit denen der Eigentümer in Einklang bringen soll. Zugleich führte der starke Fokus auf den Shareholder Value verstärkt zu einem kurzfristigen Denkansatz, der aus deutscher Sicht oft kritisiert wird. „Die Vergangenheit hat gezeigt, dass Manager hohe langfristige Risiken eingegangen sind, um kurzfristig den Gewinn und ihr daran geknüpftes variables Gehalt zu steigern“ (Jörg Rocholl in Süddeutsche Zeitung 2019). Das widerspricht dem Sicherheitsbedürfnis der deutschen Stakeholder, die eine langfristige Strategieausrichtung bevorzugen. Die Präsenz von Spitzenmanagern und Finanzexperten mit einem höheren Anteil der Vergütung von Aktienoptionen hat zur Ausweitung der Top-1-Prozent-Einkommen vor allem im angelsächsischen Raum beigetragen. Darüber hinaus gibt es unterschiedliche soziale Normen und Vorstellungen in Deutschland und den USA, was die Spreizung des Gehaltsgefüges zwischen Top Managern und Mitarbeitern betrifft (Süddeutsche Zeitung 2019; Zeit Online 2018).

## Chancen für mehr Wachstum

(1) Halten wir zunächst fest, was die Analyse zu den eingangs thematisierten Befunden einer doch sehr durchgängig erscheinenden Reserviertheit der Deutschen gegenüber Wachstum und Risiko ergeben hat:

- Die Neigung zur Sicherheit ist bei den Deutschen stark ausgeprägt. Dies äußert sich in den Erwartungen an die Finanzpolitik und an die Geldpolitik, es manifestiert sich in der entsprechenden Regulierung und Autonomisierung dieser Politikbereiche.
- Die Neigung zur Sicherheit der Deutschen zeigt sich ebenso im persönlichen Bereich, in dem das Anlageverhalten dem Ziel der Sicherheit sehr viel stärker verpflichtet ist als dem der Rendite. Die präferierten Anlagen und die dominanten Anlagestrategien tragen dem Rechnung.
- Die Renditeverluste werden nicht durch eine größere Nachhaltigkeit der privaten Investitionen in Form längerer Unternehmensgeschichten kompensiert. Sicherheit setzt auf stabile Zahlungsströme, wie sie bei dividendenstarken Aktien sowie Anleihen gegeben sind. Die Niedrigzinsphase ruft deshalb in Deutschland eine besonders harsche Kritik hervor.
- Unverändert ist Deutschland im Bereich der Risikokapitalbereitstellung eher schwach aufgestellt, im Vergleich zu den USA hat sich eine kaum zu vermindernde „Venture Capital Divide“ etabliert. In besonders risikoreiche, weil innovative Firmen in ihrer Gründungsphase zu investieren wird auch nicht durch die Hoffnung auf „high growth and high returns“ angeregt.
- Die Ursachen für diese Entwicklung greifen historisch weit zurück und sind sowohl in institutionellen Weichenstellungen als auch in kulturellen Prägungen zu finden. Zu den institutionellen Bedingungen gehören die frühe Etablierung einer Sozialversicherung, vor allem einer Alterssicherung, und die fast zeitgleich stattgefundene besondere Abkehr von der Aktienfinanzierung.
- Zu den kulturellen Prägungen zählen die Hyperinflationserfahrungen nach den beiden Weltkriegen. Die umstürzende Bedrohung des täglichen Lebens hat sich tief in das kollektive Gedächtnis der Deutschen eingeprägt und ist als kulturelle Codierung nachhaltig wirksam. Die historische Phase vom Ersten bis zum Zweiten Weltkrieg hat darüber hinaus tiefgreifend die wirtschaftlichen und gesellschaftlichen Strukturen beeinflusst.

(2) Die Frage, die sich anschließt, richtet sich auf die möglichen Ansatzpunkte für eine stärkere Mobilisierung der Kapitalbildung aufgrund von Wachstumserwartungen. Der Rückblick auf wichtige historische Ursachen der deutschen Risikoaversion hat der Robustheit entsprechender kultureller Codierung deutlich werden lassen. Auch wenn kaum noch Menschen aus eigenem Erleben über die Hyperinflation nach dem Ersten Weltkrieg und immer weniger über die nach dem Zweiten Weltkrieg berichten können, so hat doch der Generationenverbund die entsprechenden Erzählungen und deren Ikonographie beharrlich werden lassen. Wie in kaum einer anderen Gesellschaft wird in Deutschland die Verschuldung als Instrument der Staatsfinanzierung verpönt. Dabei vermengen sich mittlerweile zwei Mythen, die einer angemessenen sachlichen Unterlegung entbehren: der Mythos, dass ohne grundgesetzliche Schuldenbremse zuvor die Staatsverschuldung aus dem Ruder gelaufen sei; und der Mythos, dass die Schuldenbremse ganz wesentlichen den Konsolidierungserfolg seit dem Jahr 2009 begründet. Beides kann so nicht bestätigt werden (Hüther 2019a).

Wenn aber dem Staat, der mit hoher Glaubwürdigkeit an den Kapitalmärkten bewertet wird, kein Zutrauen mehr entgegengebracht wird, die notwendigen Zukunftsinvestitionen zu tätigen, dann wird dies in allen anderen Kontexten – Risikofinanzierung, Startup-Finanzierung, Anlageverhalten – ebenso wenig zu erwarten sein. Anders gewendet: Erst wenn der Staat zu einer anderen Perspektive auf Risiko und Wachstum findet und das entsprechend umsetzt, wird dies in der Breite zu einer anderen Einschätzung beider Kategorien führen können. Eine Strategie gezielter Investitionen für mehr Wachstum ist grundsätzlich beschrieben worden (Bardt et al. 2019; ebenso Dullien et al. 2020).

(3) Mit welchem Narrativ aber lässt sich die hierzulande festsitzende Meinung aufbrechen, dass der Staat weder mit Geld umgehen noch als innovativer Treiber fungieren könne? Es geht um den Befund zu staatlichen Innovations- und Investitionsanstrengungen. Einerseits wissen wir seit längerem aufgrund vielfältiger empirischer Studien, dass staatliche Investitionen eine hinreichend positive Ertragsrate haben und deshalb eine Kreditfinanzierung besonders dann, wenn der Zinssatz unterhalb der BIP-Zuwachsrate liegt, gut begründet werden kann (Hüther 2019a).

Noch viel grundsätzlicher aber sind die Analysen, die dazu Mazzucato (2013) vorgelegt hat. Danach hat der Staat durch die Finanzierung jener Innovationen mit hohen, unternehmerisch kaum privat zu tragenden Risiken einen zentralen Einfluss auf das gesamtwirtschaftliche Innovationsgeschehen erlangt. Der Privatsektor ist also im Bereich der grundlegenden, disruptiven Innovationen erst dann in der Lage zu investieren, wenn der Staat dieses hohe Risiko durch finanzielle Beteiligung in eine tragfähige Größenordnung transferiert hat. An vielen Einzelbeispielen – Kernenergie, Internet, Mikroprozessoren, GPS, Touchscreen, Spracherkennungssystem Siri – kann belegt werden, dass der Staat an der Finanzierung der großen Innovationen beteiligt war. Die Risikodimension solcher Neuerungen übersteigt die gewöhnliche Tragfähigkeit von Unternehmen und die Geduld der Investoren, denn die Ausreifungszeit ist in der Regel länger als sie Risikokapitalgebern hinnehmbar erscheint, jedenfalls bei den in diesen Fällen erforderlichen Beträgen, ganz abgesehen von den Erfolgsaussichten.

Damit verändert sich aber der Blick auf die Rolle des Staates in der Mobilisierung von Innovationspotenzialen. Er wird tatsächlich häufig zum Akteur der ersten Stunde und legt damit das Fundament für viele neue unternehmerische Handlungsmöglichkeiten. Das setze aber voraus, dass der Staat bereit sein muss, Ziel, Sinn und gesellschaftlichen Zweck der Innovation (mit) zu prägen, wie seinerzeit mit der Mondfahrt („mission oriented research und innovation“). Was die Analysen von Mazzucato nicht erkennen lassen, dass ist die Versagensquote öffentlicher Innovationsfinanzierung. Zu einer Gesamtschau gehört dies dazu. Deshalb wird es nicht reichen, auf die positiven Befunde zu verweisen, von denen wir erst ex post mit Sicherheit sagen können, dass diese einen beachtlichen volkswirtschaftlichen Ertrag haben.

(4) Sinnvoll erscheint eine systematische Bewertung von öffentlichen Projekten ex ante, um mit der jedenfalls möglichen Gewissheit eine bessere, überzeugendere Entscheidungsgrundlage zu gewinnen. Im Vereinigten Königreich besteht im Bereich der Infrastruktur seit dem Jahr 2016 die „Infrastructure and Projects Authority (IPA)“ als Kompetenzzentrum der britischen Regierung für Infrastrukturvorhaben und Großprojekte (Government UK 2019). Die IPA berichtet an das Kabinettsbüro und das Finanzministerium. Zu den Kernteams gehören Experten für Infrastruktur, Projektabwicklung und Projektfinanzierung, die entsprechende Nutzen-Kosten-Analysen für Infrastruktur und Großprojekte, Eisenbahnbau, Schulen, Krankenhäuser und Wohnungen bis hin zu Verteidigungs‑, IT- und großen Transformationsprogrammen.

Das kann nicht jene Projekte erfassen, für die wegen ihres disruptiv-innovativen Charakters eine Nutzen-Kosten-Abwägung gar nicht möglich ist. Hier wird es vielmehr darauf ankommen, durch ein insgesamt innovationsfreundliches Umfeld aus Forschungseinrichtungen, industrieller Verbundforschung und vielfältigen Finanzierungsmöglichkeiten dafür die grundsätzlichen Voraussetzungen zu schaffen. Offenkundig ist in den USA durch solche Einrichtungen wie DARPA („Defense Advanced Research Projects Agency“) im Zusammenspiel mit nationalen Forschungsfonds eine Förderinfrastruktur entstanden, der es grundsätzlich gelingt, neue Basistechnologien und disruptive Durchbrüche zu ermöglichen (DARPA o.J.). In Deutschland ist dem entsprechend im Jahr 2019 die „Agentur für Sprunginnovation“ gegründet worden, die „themenoffen und in einer Kultur geprägt von Risikobereitschaft, Flexibilität und Fehlertoleranz“ wirken soll (BMBF 2019). Spät folgt Deutschland damit den Erfahrungen der USA und verbindet diese mit den hierzulande etablierten Strukturen (Max Planck‑, Fraunhofer‑, Helmholtz‑, Leibniz-Gesellschaft …). Gleichzeitig gibt es in den USA bipartisan den Versuch, mit einer staatlich finanzierten Forschungsstrategie (110 Mrd. US-$) anwendungsorientiert (ähnlich Fraunhofer-Prinzip) und industriebasiert (ähnlich industrieller Verbundforschung) neue Wachstumsfelder anzustoßen – der *Endless Frontier Act* (U.S. Congress 2020).

(5) Doch all dies schafft nur Gelegenheiten. Ergriffen werden müssen die Gelegenheiten von Unternehmerinnen und Unternehmern. Das Unternehmerbild hat aber in der deutschen Öffentlichkeit seit der Finanzkrise ganz besonders gelitten. Im Zuge dessen ist die Erwartung an den Staat gestiegen und dieser wird nicht nur als Regulator, sondern zunehmend – freilich aus unterschiedlichen Gründen wie die Erfahrungen aus der Covid 19-Pandemie deutlich machen – als unternehmerischer Akteur gefordert (Röhl und Rusche 2020). So bedeutsam aber eine neue Sicht auf die Voraussetzungsqualität staatlichen Engagements gesehen wird (Mazzucato 2013), so sehr bleibt richtig, dass dies unternehmerischen Spirit, Innovationsleistung und Gründergeist erfordert. Dazu gehören auch all die bekannten Maßnahmen zu Erleichterung von Unternehmensgründungen, der regulatorischen Verbesserung für Start up-Finanzierung, die sonstigen Themen einer mittelstandsorientierten Politik (BMWi 2019).

Wie können wir das Unternehmerbild in der Öffentlichkeit verbessern? Es geht um die Frage der angemessenen Zuschreibung von Verantwortung. In einer ordnungspolitischen Perspektive wird in der marktwirtschaftlichen Ordnung nicht nach den Motiven der Akteure gefragt, da es im Wettbewerb – als Austauschprozess und als Parallelprozess – zu einer ethischen Neutralisierung des Eigennutzes als Handlungsantrieb kommt. Die Moral, so die Idee, findet sich in der Rahmenordnung und den Regelwerken verankert. Doch das reicht heute nicht mehr. Unternehmen, auch international tätige Konzerne, müssen neben der Ergebnisverantwortung ebenso Reputationsverantwortung und Ordnungsverantwortung gerecht werden. Die Ergebnisverantwortung bringt das einzelwirtschaftliche Kalkül zum Ausdruck, die Reputationsverantwortung (Wahrnehmung im öffentlichen Raum) sowie die Ordnungsverantwortung (Mitgestaltung der relevanten Regelwerke und Institutionen) greifen weit darüber hinaus und lassen sich als gesellschaftliche Verantwortung kategorisieren (Hüther 2019b).

Unternehmen werden damit als soziale Errungenschaft moderner Gesellschaften verstanden, mit der durch Kooperationsleistungen effizient und wirksam individuelle, kollektive und gesellschaftliche Probleme gelöst werden. Die gesellschaftliche Verantwortung von Unternehmen erweist sich als eine sehr komplexe Struktur, die sich nicht nur in der Werteorientierung des Unternehmens, seiner definierten Mission und gelebten Kultur spiegelt, sondern ebenso in der Aufbau- und der Ablauforganisation – der inneren Verfasstheit (Hüther 2019b, S. 446): „Denn mit zunehmender betriebswirtschaftlicher Integrationstiefe an ausländischen Standorten durch Direktinvestition nimmt das Interesse der dortigen Zivilgesellschaft an dem Unternehmen zu und entwickelt sich zu einer veritablen Anspruchsgruppe. Gleichzeitig aber muss der Heimatstandort aufgrund seiner historischen Wurzeln mit zunehmender transnationaler Unternehmensaufstellung die Ankerfunktion für die Unternehmenskultur ausprägen. Daraus folgt, dass die gesellschaftliche Verantwortung – Reputationsverantwortung und Ordnungsverantwortung – des Unternehmens im Heimatstandort definiert wird und damit zugleich Orientierung für die Reputation auf den ausländischen Märkten schafft, während die Ergebnisverantwortung auf allen Märkten gleichermaßen greift.“

## Footnotes

1 Trotz niedriger Zinsen stieg der Anteil der Sichteinlagen an den Bankguthaben privater Haushalte 2019 auf 62,8 %. Am gesamten Geldvermögen der privaten Haushalte machen Bargeld und Sichteinlagen inzwischen 26,7 % aus.

2 Für die Jahre 2006–2018; kleine und mittlere Unternehmen mit einem Umsatz von bis zu 500 Mio. € pro Jahr (KfW 2019).

3 Die aktuelle Entwicklung der Dividendenzahlungen in volatilem Kursumfeld bestätigen diesen Zusammenhang, vgl. Handelsblatt (2019).
